# Application of Virtual Reality, Augmented Reality, and Mixed Reality in Endourology and Urolithiasis: An Update by YAU Endourology and Urolithiasis Working Group

**DOI:** 10.3389/fsurg.2022.866946

**Published:** 2022-04-01

**Authors:** B. M. Zeeshan Hameed, Shraddha Somani, Etienne Xavier Keller, R. Balamanigandan, Satyasundara Mahapatra, Amelia Pietropaolo, Şenol Tonyali, Patrick Juliebø-Jones, Nithesh Naik, Dilip Mishra, Sarvesh Kumar, Piotr Chlosta, Bhaskar K. Somani

**Affiliations:** ^1^Department of Urology, Father Muller Medical College, Mangalore, India; ^2^European Association of Urology—Young Academic Urologists Urolithiasis and Endourology Working Group, Arnhem, Netherlands; ^3^International Training and Research in Uro-oncology and Endourology Group, Manipal, India; ^4^Department of Computer Science and Engineering, Manipal Institute of Technology, Manipal Academy of Higher Education, Manipal, India; ^5^Department of Urology, University Hospital Zurich, University of Zurich, Zurich, Switzerland; ^6^Department of Artificial Intelligence, Institute of Computer Science and Engineering, Saveetha School of Engineering, Saveetha Institute of Medical and Technical Sciences, Saveetha University, Chennai, India; ^7^Computer Science and Engineering, Pranveer Singh Institute of Technology, Kanpur, India; ^8^Department of Urology, University Hospital Southampton National Health Service (NHS) Trust, Southampton, United Kingdom; ^9^Department of Urology, Istanbul University Istanbul Faculty of Medicine, Istanbul, Turkey; ^10^Department of Urology, Haukeland University Hospital, Bergen, Norway; ^11^Department of Clinical Medicine, University of Bergen, Bergen, Norway; ^12^Department of Mechanical and Manufacturing Engineering, Manipal Institute of Technology, Manipal Academy of Higher Education, Manipal, India; ^13^Department of Urology, Global Rainbow Healthcare, Agra, India; ^14^Department of Computer Science and Engineering, Babu Banarasi Das University, Lucknow, India; ^15^Department of Urology, Jagiellonian University in Kraków, Kraków, Poland

**Keywords:** virtual reality, augmented reality, mixed reality, endourology, urolithiasis (urinary stones)

## Abstract

The integration of virtual reality (VR), augmented reality (AR), and mixed reality (MR) in urological practices and medical education has led to modern training systems that are cost-effective and with an increased expectation toward surgical performance and outcomes. VR aids the user in interacting with the virtual environment realistically by providing a three-dimensional (3D) view of the structures inside the body with high-level precision. AR enhances the real environment around users by integrating experience with virtual information over physical models and objects, which in turn has improved understanding of physiological mechanisms and anatomical structures. MR is an immersive technology that provides virtual content to interact with real elements. The field of urolithiasis has adapted the technological advancements, newer instruments, and methods to perform endourologic treatment procedures. This mini-review discusses the applications of Virtual Reality, Augmented Reality, and Mixed Reality in endourology and urolithiasis.

## Introduction

The surgical industry seeks out modern and cost-effective training systems with an increased expectation toward surgical performance and outcome with greater reactivity to medico-legal considerations (1, 2). Recent advancements in technologies have led to the integration of Virtual Reality (VR), Augmented Reality (AR), and Mixed Reality (MR) with medical tools, equipment, and training kits into medical education. The simulation in surgical education and healthcare applications is strongly influenced by industries like aviation, automobile, the military for real-world training, and experience (3, 4). The studies and investigations from the literature validate that simulation training for medical students and residents to prepare for hands-on procedures has become an effective and reliable approach (5, 6).

VR aids the user in interacting with the virtual environment realistically by providing a three-dimensional (3D) view of the structures inside the body with high-level precision. VR is proven to be an effective method of training students and residents alike with hands-on procedures due to the recent advancements made in haptics, high-resolution audio-visual effects, motion detection, and display systems. With the use of VR, skills like suturing can be practiced on robotic consoles, simulation tools can recreate mid-stages of surgeries with high and low accuracy dry labs, animal models, wet-lab cadaveric organs, thus providing an optimal experience and learning (1–5).

**Figure 1:**
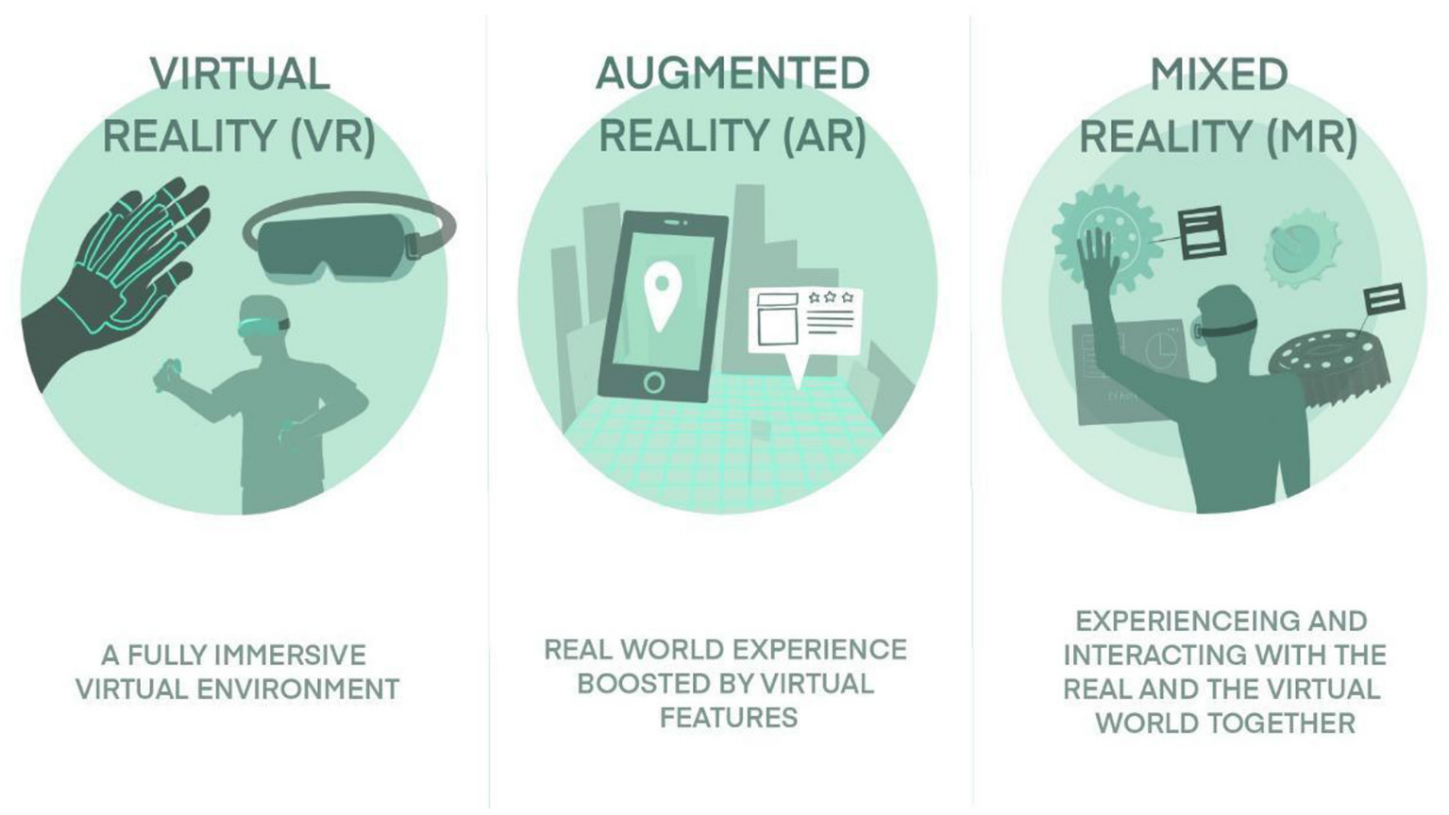
Differentiation of immersive experience (Virtual Reality, Augmented Reality, and Mixed Reality) medical simulation tools.

AR enhances the real environment around users by integrating experience with virtual information over physical models and objects, which in turn has improved understanding of physiological mechanisms and anatomical structures (1). Simulation-based training can be performed in isolation or a “full immersion simulation” where operating conditions are mimicked for maximum experience to increase technical and non-technical skills in return for improved patient results and team performance (7). MR is the most recent form of immersive technology, wherein the virtual contents interact with real elements. This is typically achieved by translucent glasses on which the virtual content is projected. MR requires considerably more sensors and processing power in comparison with VR and AR. Figure 1 shows the differentiation of immersive experience (Virtual Reality, Augmented Reality, and Mixed Reality) medical simulation tools.

An endoscopic method or examination executed using a percutaneous approach from the urethra to the kidney is referred to as “endourology” (17). The traditional “hands-on” training approach does not lend itself well to minimal access surgery, which results in a fragmented approach to surgical education that lacks consistency and is both time and labor-intensive. Thus, paving a path for exploring newer technologies that aid improved experience in clinical practices, surgical education, patient counseling, diagnosis, and treatment. The field of urolithiasis and endourology has progressed immensely in the past three decades and has adapted the technological advancements, newer instruments, and methods to perform endourologic treatment procedures.

## Role of VR/AR/MR in Endourology and Urolithiasis

Renal stone is a common urological disease encountered irrespective of gender, ethnicity, or age (18). Percutaneous nephrolithotomy (PCNL) is a crucial component of the treatment of renal stones and is the most developed procedure for large or numerous kidney stones. With the growth of technology, procedures like stone removal treatments have become more technologically driven (18). PCNL delivers stone-free rates of over 90% while significantly reducing the risk of complications due to advanced technological equipment (19–21). Percutaneous renal access (PCA) is one of the hardest steps during PCNL since the kidney is surrounded by very important body parts, such as the spleen, the colon, and the liver which can be accidentally punctured while trying to establish the kidney due to inaccurate needle placement and insertion. Studies conducted in the past reported that almost 70% of the percutaneous procedures were performed by practicing urologists, however, only 11% could achieve PCA themselves with the reason stated as the lack of practice during training (22–25).

The PERC Mentor is a VR simulator specially developed for percutaneous kidney access training. The study included 56 participants to test the varying validity of VR training for PCA. During the study, a standardized questionnaire was submitted to the expert group to evaluate the validity of the simulation. The beginner group received two 30 min supervised training sessions on the PERC Mentor to facilitate learning of the percutaneous kidney access skill (8). A total of 24 participants were fully evaluated who completed the process including two cohorts of 15 beginners and 9 experts (clinical experience of performing PCNL in more than 50 cases). A subgroup of five beginners underwent training on pigs before PERC Mentor training. Five beginners (who initially performed the task on the pig) repeated the same task on the pig. After the PERC mentor training, they showed a statistically significant improvement in the reduction in total surgical time.

The ideal teaching aid for beginners is manual training on living patients which unfortunately carries various ethical issues. The ease by which basic endourological procedures like PCA could be performed using virtual reality simulators resulted in improved operating room performance. This study has also concluded that simulation can be used to refine techniques and tactics of new medical students. Table 1 lists the recent studies and their outcomes related to the application of virtual reality (VR), augmented reality (AR), and mixed reality (MR) in endourology and urolithiasis.

**Table 1:** Summary of studies related to the application of virtual reality (VR), augmented reality (AR), and mixed reality (MR) in endourology and urolithiasis.

**References**	**Objective**	**Tool used**	**Method**	**Results**
Mishra et al. (8)	Validity and performance testing of virtual reality-based training for PCA	PERC Mentor	To test the varying validity of VR training for PCA −56 participants	Beginners with PERC Mentor training showed statistically significant improvement in the reduction of total surgical time during PCA
			Participants fully evaluated and completed the process −24 participants	
			Two cohorts: 15 beginners and 9 experts	
			Five beginners were trained on pigs before PERC Mentor training	
Matsumoto et al. (9)	Testing and performance comparison Symbionix model versus high fidelity ureteroscopy guide model	Uro Mentor	Sixteen residents in urology were evaluated on their competence to execute various tasks on a VR simulator	Senior residents scored higher (statistically significant) and overall took less time to complete the task in comparison to the junior residents
			The evaluation was based on a global rating scale, and a Pass/Fail rating to evaluate the subject's performance	The tool is good to assess the skills of surgical residents
Raison et al. (10)	Skill assessment of urology postgraduate trainees in percutaneous renal access (PCA)	PERC Mentor	Objective Structured Clinical Examinations (OSCEs) to study the impact of previous percutaneous nephrolithotomy (PCNL)	The postgraduate trainees with previous experience in PCNL performed significantly better and faster
Knudsen et al. (11)	Evaluate and establish face, content and construct validation of the PERC Mentor simulator	PERC Mentor	Total 63 participants were divided into two groups: (a) Intervention group (underwent two 30-min training sessions on the simulator) (b) Control group (no further training)	Intervention group participants had improved and better performance
Nayahangan et al. (12)	Integration of urological procedures into simulation-based training for resident trainees	–	The Delphi method was used to conduct a national needs assessment	The qualified experts have chosen in three rounds created and developed a simulation-based training program for the new urologists
			The study involved a total of 56 experts with significant roles in urology education	
Aydin et al. (13)	Evaluating current training methods and soliciting feedback on the potential role of AR simulation in urological training	–	A cross-sectional survey containing three sections: (a) Introduction (b) Technical skills in urology (c) Non-technical skills in urology	Both trainees and specialists advocate simulation, as the solution for safe and effective urological procedural training
Hu et al. (14)	Comparison of post-training ureteroscopy and cystoscopy competency	Uro-scopic trainer	The study involved 36 participants divided into three groups, was assessed on the Objective Structured Assessment of Technical Skills (OSATS) scale: (a) Trained with the transparent simulator (b) Trained with the non-transparent simulator (c) Trained with verbal instructions	Students improved their ureteroscopy and cystoscopy proficiency with simulator training
	Unique transparent anatomic simulator vs. no simulator training			Transparent simulators were more successful than other methods
Cai et al. (15)	Investigating the effectiveness of VR simulator training in the treatment of kidney stones using retrograde flexible ureteroscopy	Uromentor	Participants underwent 4-h training and practice sessions on VR simulators	Significant improvement (*P* < 0.01) made by trainees in procedure times, etc., after training on the VR simulators
			The participants were assessed on procedure time, techniques, and ability to perform specific tasks	
Zhang et al. (16)	Validating the use of PERC Mentor in percutaneous renal access training	PERC mentor	Total participants −21 urologists	Participants who had simulation-based training performed considerably faster
			The instructional video was shown, then the PERC Mentor was used to conduct percutaneous renal access	VR simulator offers high-quality training to accurately assess trainees' abilities in fluoroscopy-guided PCA
			Participants were judged based on the global rating scale	

Symbionix's Uro Mentor^TM^ is a virtual reality ureteroscopy simulator that can potentially support the teaching and evaluation of surgical residents. Matsumoto et al. (9) conducted a study in which 16 residents were evaluated on their competence to execute various tasks like guidewire insertion, performing cystoscopy, and extraction of a ureteric stone on a VR simulator. A blinded checklist was used by the examiner with a global rating scale, and a Pass/Fail rating to evaluate the subject's performance. Computer-generated characteristics such as task completion time, scope and instrument trauma, and the attempts to introduce a guidewire were also examined. A performance comparison between the high-fidelity ureteroscopy bench model and VR simulator was carried out. The senior residents trained on the VR simulator, scored considerably higher on their global rating scale, checklist, pass/fail rating, and required a significantly lesser time to complete the task whereas the junior residents showed a higher rate of trauma than senior residents. A ureteroscopy simulator proved to be a great tool for evaluating endourological skills in residents.

Noureldin et al. conducted a study to evaluate the skills of urology postgraduate trainees in percutaneous renal access (PCA) using Objective Structured Clinical Examinations (OSCEs) to observe the impact of previous percutaneous nephrolithotomy (PCNL) experience on outcomes. A brief questionnaire was used to assess the previous experience of trainees in endourology. The PERC Mentor^TM^ is a device that is used to teach the operator how to perform the percutaneous renal collecting system access puncture. After a 3-min briefing on this simulator at Simbionix, Cleveland, OH, the trainees then demonstrated their ability to access the renal calyces in a model of a normal left kidney and pop the balloons present. The data collected from the completed simulations' performance report and the questionnaires were thoroughly analyzed. The PERC Mentor simulator can be used to examine the PCA abilities of urology trainees during OSCEs. The trainees with prior PCNL knowledge performed better and had fewer problems (10).

Knudsen et al. conducted a study that included 63 trainees to evaluate and establish face, content and construct validation of the PERC Mentor simulator. The subjects were then randomly assigned to one of two groups: intervention (underwent two 30-min training sessions on the simulator) or control (no further training). When compared to their baseline performance and the untrained control group, subjects who received simulator training showed significant improvements in objective and subjective parameters. The study concluded that training on simulators may enable trainees to gain the fundamental skills required for PCA since practicing on live models always comes with a handful of ethical restrictions and complications (11).

The study conducted by Nayahangan et al. (12) aimed to incorporate urological procedures into simulation training conducted during the residency period. Delphi method was used to conduct a national wide need assessment involving 56 experts having significant roles in urology education. The assessment was carried out in three rounds wherein, Round 1 involved sorting the relevant procedures to decide the tasks that can be performed by the newly qualified urologists. Round 2 involved investigations wide a survey with a need assessment formula to identify the following: procedure frequency, how important the procedure is and how many physicians should be able to carry it out, the patient response and associated risks when a procedure is performed by a beginner physician, and the feasibility of simulation training. The ranking based on the importance of procedure, elimination of unsuitable candidates was carried out in Round 3. Cystoscopy, transrectal ultrasound-guided prostate biopsy, ureteral stent placement, urethral and suprapubic catheter insertion, and transurethral resection of the bladder were the five urological procedures with the highest priority that qualified to be converted to simulations to create and develop a training program for the new urologists (12).

Aydin et al. evaluated the effectiveness and limitations of simulation in urology training and assessment. Types of simulations (synthetic, VR, and animal models) with participant experience levels and the number of tasks completed were considered in the assessment process. For the early stage of training and testing, current simulation tools are credible and accurate. Modalities can be used to teach intermediate and expert level techniques, but their availability is limited due to supply shortages and ethical concerns. Over the last few decades, several medical institutes have readily accepted simulation-based training as a supplement to conventional operating theater experience for improved technical and non-technical skill training (13).

Huet al. (14) compared post-training ureteroscopy and cystoscopy competency with a unique transparent anatomic simulator, an opaque model, vs. no simulator training. Ten experienced urologists conducted a preliminary review of the models as teaching materials. Thirty-six first-year medical students who received the same theoretical training, were rated on a 50-point scale on their theoretical knowledge. The students were placed into three groups: those who received training with a transparent simulator (Group 1), those who received training with a non-transparent simulator (Group 2), and those who only received comprehensive verbal instruction (Group 3). The trainee's ability to insert and remove ureteral stents was assessed using the Uro-scopic Trainer and rated on an Objective Structured Assessment of Technical Skills (OSATS) scale after 12 days of training. All 10 urologists who evaluated the devices agreed that they were anatomically correct, that either version was simple to use, and that they were good ureteroscopy and cystoscopy training tools. Students improved their ureteroscopy and cystoscopy skills with simulator training, and transparent simulators were shown to be more effective than their counterparts (14).

The effectiveness of virtual reality simulator training in the treatment of kidney stones using retrograde flexible ureteroscopy was investigated by Cai et al. The results revealed a considerable improvement in the management of renal stones using retrograde flexible ureteroscopy after completing the 4-h special-purpose training using VR simulators. Between the first and second assessments, there were several statistically significant differences (*P* < 0.01). Finally, the virtual reality simulator training program can assist trainees in quickly improving their retrograde flexible ureteroscopy skills for the treatment of renal stones (15).

Zhang et al. conducted a study to establish the effectiveness of the PERC Mentor simulator in percutaneous renal access training. A fluoroscopy-guided percutaneous kidney accessing technique was introduced to 21 urologists. Ten of the 21 students had never performed percutaneous nephrolithotomy under ultrasound guidance earlier. Thus, the trainees were divided into two groups: those with primitive experience and those with no experience. When comparing the primitive experience group to the inexperienced group, the amount of contrast material used, and overall operating time were significantly lower in the primitive experience group (*P* = 0.03 and 0.02, respectively). The PERC Mentor simulator allows trainees with no prior expertise in fluoroscopy-guided PCA to complete the virtual manipulation of the process independently. This VR simulator is essential for offering high-quality training and may be used to accurately assess trainees' abilities in fluoroscopy-guided PCA (16).

Checcucci et al. evaluated surgeons' perception of mixed reality for partial nephrectomy. Attendees were given the opportunity to try MR for themselves and share their opinion on its application using a Likert scale (1–7, 17–19) questionnaire. A total of 172 participants shared their opinion. Both the surgical planning and anatomical accuracy scores (8 and 9, respectively) were excellent. This technology's potential role in preoperative planning and comprehension of surgical complexity (both rated 9/10) was expressed with high satisfaction by the participants. A more selective approach was chosen by 64.4% of surgeons and 44.4% after using HoloLens and MR technology for the first time in the field of surgery instead of just using CT images for guidance. According to the findings of the study, surgeons believe holograms and MR imaging to be a useful and interesting preoperative tool before partial nephrectomy (26).

Checcucci et al. summarized the most recent research on PCNL's use of virtual imaging guidance. Surgery training and surgical planning in urology were the first applications for PCNL 3D virtual navigation technology, which was later expanded into the field of surgical navigation using various modalities. Tools that focus on surgery have proven to be beneficial to both surgical planning and surgical navigation by using augmented or mixed reality systems that assist the surgeon in real time during an intervention (27).

Francesco Porpiglia et al. evaluated the feasibility of 3D MR holograms for establishing the point of access and directing the needle during percutaneous kidney puncture. Ten patients underwent 3D MR endoscopic combined intrarenal surgical procedure (ECIRS) for kidney stones were included in the study. A matched pair analysis was performed on a group of patients who had previously undergone a standard procedure. Different patient characteristics were compared between groups prior to and following surgery. Statistical tests for continuous and categorical variables was performed. Using 3D MR guidance for renal puncture is safe and effective, according to the study results. As a result of the MR guidance, the inferior calyx was punctured correctly in all cases, and the procedure was found to be safe and effective (28).

## Conclusion

Numerous methods are introduced and practiced by students in medical education to make their learning and practice easier before hands-on experience. Researchers have cumulatively agreed that simulation-based training as being one of the effective modalities for teaching and training. AR in medicine enables trainees to experience full operating conditions, but VR and MR allow them to practice and improve on skills like suturing, which otherwise would be performed on animal models and raise various ethical issues. Additionally, tools like the PERC Mentor and Uromentor have shown a remarkable impact in the field of endourology with several studies evaluating and proving their effectiveness. The results have proven that students often perform better after being trained on these simulators. Moving forward, surgical education is bound to improve as medical technology advances and simulation-based training becomes a permanent and vital part of a medical student's curriculum.

## Future Directions

Several studies have been conducted on the application of VR/AR in urology, however, they have only been limited to training simulators and performing surgery. Their clinical use in endourology has been limited to pilot studies in PCNL puncture but a wider adoption of this is still lacking in the clinical field of endourology. These simulators have usages such as flexible ureteroscopy, laparoscopic surgery, robotic surgery, and detecting prostate cancer. While these applications have been proven useful, to further advance the field of urology, usage of AR/VR must broaden as well. AR/VR technology can be used to reflect symptoms of patients when they are going on with their daily lives—not only would this give a greater insight to the assigned doctor, but the treatment will have high accuracy and be more effective. To conclude, the growing field of AR/VR technology has opened a door to various opportunities to improve patient care in endourology.

## Author Contributions

BH, NN, AP, and BS contributed to the conception and design of the study. EK, ŞT, AP, PJ-J, and DM organized the database and wrote the first draft of the manuscript. NN, SK, ŞT, RB, SM, and BH wrote sections of the manuscript. PC, AP, EK, and BS critically reviewed and edited the manuscript. All authors contributed to manuscript revision, read, and approved the submitted version.

## Conflict of Interest

The authors declare that the research was conducted in the absence of any commercial or financial relationships that could be construed as a potential conflict of interest.

## Publisher's Note

All claims expressed in this article are solely those of the authors and do not necessarily represent those of their affiliated organizations, or those of the publisher, the editors and the reviewers. Any product that may be evaluated in this article, or claim that may be made by its manufacturer, is not guaranteed or endorsed by the publisher.
